# Role of Lipid Peroxidation Products, Plasma Total Antioxidant Status, and Cu-, Zn-Superoxide Dismutase Activity as Biomarkers of Oxidative Stress in Elderly Prediabetics

**DOI:** 10.1155/2014/987303

**Published:** 2014-05-05

**Authors:** Sylwia Dzięgielewska-Gęsiak, Ewa Wysocka, Sławomir Michalak, Ewa Nowakowska-Zajdel, Teresa Kokot, Małgorzata Muc-Wierzgoń

**Affiliations:** ^1^Teaching Department and Hospital Ward of Internal Diseases, Medical University of Silesia, Żeromskiego 7 Street, 41-902 Bytom, Poland; ^2^Department of Clinical Biochemistry and Laboratory Medicine, Poznań University of Medical Sciences, Grunwaldzka 6 Street, 61-702 Poznań, Poland; ^3^Department of Neurochemistry and Neuropathology, Poznan University of Medical Sciences, Przybyszewskiego 49 Street, 60-355 Poznań, Poland; ^4^Neuroimmunology Unit, Polish Academy of Sciences, Przybyszewskiego 49 Street, 60-355 Poznań, Poland

## Abstract

The relationship between hyperglycemia and oxidative stress in diabetes is well known, but the influence of metabolic disturbances recognized as prediabetes, in elderly patients especially, awaits for an explanation. *Methods*. 52 elderly persons (65 years old and older) with no acute or severe chronic disorders were assessed: waist circumference (WC), body mass index (BMI), percentage of body fat (FAT), and arterial blood pressure. During an oral glucose tolerance test (OGTT) fasting (0′) and 120-minute (120′) glycemia and insulinemia were determined, and type 2 diabetics (*n* = 6) were excluded. Subjects were tested for glycated hemoglobin HbA1c, plasma lipids, total antioxidant status (TAS), thiobarbituric acid-reacting substances (TBARS), and activity of erythrocyte superoxide dismutase (SOD-1). According to OGTT results, patients were classified as normoglycemics, (NGT, *n* = 18) and prediabetics, (PRE, *n* = 28). *Results*. Both groups did not differ with their lipids, FAT, and TBARS. PRE group had higher WC (*P* < 0.002) and BMI (*P* < 0.002). Lower SOD-1 activity (*P* < 0.04) and TAS status (*P* < 0.04) were found in PRE versus NGT group. *Significance*. In elderly prediabetics, SOD-1 and TAS seem to reflect the first symptoms of oxidative stress, while TBARS are later biomarkers of oxidative stress.

## 1. Introduction 

In the XXI century an elderly population (65 years old and older) will grow [1, 2]. Studies on obesity, hypertension, dyslipidemia, and hyperglycemia in elderly population are currently widely discussed [3, 4]. Patients with type 2 diabetes mellitus suffer from late diabetic complications—atherosclerosis, hypertension, and dyslipidemia [5–7]. Chronic hyperglycemia leads to oxidative stress, dyslipoproteinemia, glycation of proteins, and endothelial dysfunction [8–10].

Over 40% of those affected by carbohydrate metabolism disturbances are 65 or older [11]. Among elderly subjects the late diabetic complications are more common. Many studies have been carried out to evaluate markers of free radical-induced lipid peroxidation and antioxidant status in diabetic patients [12]. Thus, we know how diabetic hyperglycemia influences oxidant-antioxidant stress parameters [13], but still it is not clear in which way prediabetic hyperglycemia may influence metabolic balance in elderly patients. The oxidative stress may accompany and explain metabolic complications in hyperglycemic persons [14].

Hence, the present study has been undertaken, to evaluate the hypothesis that oxidative stress in elderly patients with increased risk for diabetes (prediabetes, impaired fasting glucose, IFG, and impaired glucose tolerance, IGT) is responsible, at least partially, for the clinical and metabolic complications. There is some evidence of early symptoms of cell damage caused by acute short-time elevated glucose concentration in medium, since changes of the NAD^+^/NADH ratio, mitochondrial membrane potential, and reactive oxygen species production were observed in human hepatic carcinoma model exposed to hyperglycemia-like* in vitro* situation [15].

An incomplete reduction of oxygen to water during electron transport chain in mitochondria is a possible source of oxygen-free radicals, that is, superoxide radical ^∙^O_2_^−^, in the elementary model of oxygen-free radical production. The following oxidative modification of biomolecules is discussed in many pathologies. The human body presents natural defense against free radicals: antioxidants preventing the formation of free radicals (i.e., metal-binding proteins), antioxidants scavenging free radicals and derivates: enzymatic (i.e., superoxide dismutase, catalases, glutathione peroxidase, and paraoxonase) and nonenzymatic (including vitamins, uric acid, bilirubin, and proteins) systems, and repair enzymes (targeting DNA especially). Cooper and zinc-containing superoxide dismutase, Cu-, Zn-SOD (SOD-1), cytoplasmic enzyme, metabolizes superoxide radicals to molecular oxygen and hydrogen peroxide providing defence against oxygen toxicity [16].

The low-weight molecules are involved in the total plasma antioxidant status in the following proportions: 35–65% for uric acid, 10–50% for plasma proteins, 14% for vitamin C, and 7% for vitamin E [17], while other investigators assessed the detailed contribution of thiol groups (52.9%), uric acid (33.1%), vitamin C (4.7%), bilirubin (2.4%), vitamin E 1.7%, and others (5.2%) [18]. Researchers agree that due to the participation of many factors in the creation of plasma antioxidant defense and their possible variability, in pathological conditions of a significant share of oxidative stress, the total measurement could be more valuable [18]. Thus, intracellular antioxidant enzyme, the erythrocyte Cu-, Zn-superoxide dismutase (SOD-1), and the plasma total antioxidant status (TAS) as extracellular were chosen to describe the antioxidant potential. Thiobarbituric acid-reacting substances (TBARS) were to reflect plasma lipid peroxidation products.

## 2. Material and Methods

The study was performed under the permission from local ethics group in accordance with the Declaration of Helsinki of 1975 for Human Research and the study protocol was approved by the Bioethics Committee of Poznan University of Medical Sciences in Poznan, Poland (statements numbers 142/11 and 595/11). The subjects participating in the study gave informed consent to the study procedure.

### 2.1. Subjects

This study enrolled 313 elderly Caucasians (65 years old and older) with no complains, from Poznan metropolitan area (west of Poland). Nonsmoking elderly persons, using no medication, no special diet, no supplements, and no alcohol, without acute or chronic disease, were studied. The exclusion criteria were the positive history of stroke, coronary artery disease (accompanied by current steady-state electrocardiography), diabetes, neoplastic disease, and inflammatory disease. Additional biochemical exclusion criteria were albuminuria reflected by albumin/creatinine ratio >30 mg of albumin/1 g of creatinine in fresh morning urine sample and decreased eGFR (less than 60 mL/min) based on MDRD formula eGFR (mL/min/1.73 m^2^) = {186 × [creatinine]^−1.154^× [age]^−0,203^× 0.742 [for women] × 1.210 [for Afro-American]}. Complete physical examination, including the measurement of waist circumference (WC), systolic (SBP) and diastolic (DBP) arterial blood pressure, percentage of body fat (FAT) measured by bioimpedance method using BodyStat equipment, and the calculation of body mass index (BMI = kg/m^2^), was performed.

Finally 52 individuals were qualified for the 75 g oral glucose tolerance test (OGTT) due to WHO recommendations [19]. Results of OGTT allowed classifying subjects for normal glucose tolerance (NGT) (*n* = 18, mean age 69.0 years) and prediabetic (PRE) (*n* = 28, mean age 71.0 years) categories, while newly diagnosed type 2 diabetes mellitus (T2DM, *n* = 6) patients were excluded from the study. The interpretation of oral glucose tolerance test is presented in Table 1 [20].

### 2.2. Blood Sampling and Biochemical Analysis

Blood was collected from the ulnar vein twice: fasting at 0 min (0′) and at 120 min (120′) of the 75 g OGTT. Fasting blood sample was used to measure the level of glycated hemoglobin (HbA_1c_) as well as glucose, insulin, and lipid concentrations in plasma samples without symptoms of hemolysis. Oxidant-antioxidant balance was measured in fasting blood samples. Blood collected at 120 min of OGTT was used for plasma glucose and insulin determinations.

#### 2.2.1. Glucose, Lipid, Insulin, and HbA_1c_ Assays

Oral glucose tolerance test was performed according to WHO recommendations between 7.00 and 9.00 am. Glucose concentrations were determined at 0 minutes and 120 minutes of OGTT, following a standard dose of 75 g glucose load. Glucose and lipid parameters, including total cholesterol (T-C), high density lipoprotein cholesterol (HDL-C), and triacylglycerols (TAG) concentrations, were evaluated by enzymatic methods using bioMerieux reagent kit (Marcy-l'Etoile, France) and the UV-160A Shimadzu spectrophotometer (Shimadzu Co., Kyoto, Japan). Low density lipoprotein cholesterol (LDL-C) was calculated using the Friedewald formula for lipid parameters expressed in mmol·L^−1^: [LDL-C] = [T-C] − [HDL-C] − [0.45·TAG], if TAG <4.56 mmol·L^−1^.

Insulin concentration was measured by an ELISA method (BioSOurce, Nivelles, Belgium) with sensitivity of 0.15 mU*·*L^−1^, using microplate reader Sunrise (Tecan Group, Männedorf, Switzerland). The intra- and interassay coefficients of variation (CV) were 3.8% and 4.5%, respectively.

Glycated hemoglobin (HbA_1c_) level was determined by ion exchange high performance liquid chromatography using D-10 Instrumentation (BioRad, Heidelberg, Germany) due to the national glycohemoglobin standardization program (USA), with the sensitivity 0.05% of HbA_1c_, and intra- and interassay CV for HbA_1c_ measurement were 5.0% and 6.8%, respectively.

*The Reference Sera*. RANDOX Assayed Human Multi-Sera Level 1 (as normal) and RANDOX Assayed Human Multi-Sera Level 2 (as pathological) (Randox, Crumlin, United Kingdom) were used for monitoring the accuracy of the determinations.

#### 2.2.2. Oxidative Stress Markers

Concentration of plasma total antioxidant status (TAS) and activity of erythrocyte cytoplasmatic superoxide dismutase Cu-, Zn-SOD (EC: 1.15.1.1) (SOD-1) were measured spectrophotometrically by a colorimetric assay based on the decrease of the optical density of the blank produced by each sample in analogy to its antioxidant property using Randox reagent kits (Randox Laboratories Ltd., Crumlin, Co. Antrim, United Kingdom) and Stat Fax 1904 Plus spectrometer (Awareness Technology, Inc., Palm City, Florida, USA).

*Total Antioxidant Status (TAS)*. Total antioxidant status was carried out using ABTS^+^ (2,2′-azino-bis(3-ethylbenzothiazoline-6-sulphonic acid) radical formation kinetics. The presence of antioxidants in plasma suppressed the bluish-green staining of the ABTS cation, which was proportional to the antioxidant concentration level. Kinetics was measured at 600 nm. The intra- and interassay CV for plasma TAS concentrations were 1.5% and 3.8%, respectively.

*Red Blood Cell Cu-, Zn-Superoxide Dismutase (SOD-1) EC: 1.15.1.1..* The method employs xanthine and xanthine oxidase (XOD) to generate superoxide radicals, which react with 2-(4-iodophenyl)-3-(4-nitrophenol)-5-phenyltetrazolium chloride (INT) to form a red formazan dye. SOD-1 activity was measured by degree of inhibition of the reaction. Kinetics was measured at 505 nm. The intra- and interassay CV for SOD-1 were 1.6% and 2.7%, respectively.

*Thiobarbituric Acid-Reacting Substances (TBARS)*. Concentration of plasma TBARS, reflecting plasma lipid peroxidation products, was determined by Okhawa method [21] using Sigma reagents (Germany) and Specord M40 spectrometer (Germany). The intra- and interassay CV for TBARS were 1.8% and 3.7%, respectively.

### 2.3. Statistical Analysis

Statistica 10.0 version for Windows was used for statistical analysis. The normality of value distribution was checked by Shapiro-Wilk test. Then, the results with a Gaussian distribution were analyzed with Student's *t*-test, and those with a non-Gaussian distribution were verified by a nonparametric Mann-Whitney *U* test to assess the differences between studied NGT and PRE groups. The Spearman rank correlation test was used to evaluate the strength of association between two variables. Multiple regression analysis was performed to evaluate the relationship between independent variables and SOD-1 activity and TAS and TBARS concentrations. A *P* < 0.05 was taken as indicative of significant differences. The results with a Gaussian distribution are expressed as mean and standard deviation (SD), and those with a non-Gaussian distribution are expressed as median and interquartile range.

The correlations between studied oxidative stress markers (TBARS, SOD-1, and TAS) and age, BMI, waist circumference, FAT, plasma lipids, HbA1c, G0′, and G120′ were tested with the use of multiple regression comparison. The analyzed models included the following:

- age, BMI, waist circumference (WC), and percentage of body fat (FAT) (Table 4);
- HbA1c, G0′, and G120′ (Table 5);
- SBP, DBP, age, BMI, FAT, and WC (Table 6);
- T-C, HDL-C, and TAG (LDL-C was not included as derivative of analyzed variables).

Analyzed subgroups of patients were as follows:

- females/males;
- AH = 0/AH = 1, as with no arterial hypertension (AH = 0) and with arterial hypertension (AH = 1);
- G0′ = 0/G0′ = 1, as with no fasting hyperglycemia (G0′ = 0) and with fasting hyperglycemia (G0′ = 1);
- PRE = 0/PRE = 1 as with no prediabetes (PRE = 0) and with prediabetic states IFG or IGT (PRE = 1).

## 3. Results

The data in Table 2 show oxidative-antioxidative status and the clinical and biochemical characteristics of the groups. In the study population, prediabetes was diagnosed according to American Diabetes Association Standards for Medical Care 2013 [20] using OGTT and reflected 63.0% of IFG and 37.0% of IGT. Normoglycemic and prediabetes groups did not differ in lipid profile and percentage of body fat, but PRE group had higher waist circumference (*P* < 0.002) and BMI (*P* < 0.002). Concerning the oxidative stress markers, decreased SOD-1 (*P* = 0.033) and TAS (*P* = 0.039) and increased TBARS (no significance, *P* = 0.062) were observed in the elderly prediabetics.

Correlation analysis considering oxidative stress markers and other parameters, in both groups, was performed (Table 3). In normoglycemic elderly subjects highly positive correlation between TAS and SOD was observed, whereas such an association was not found in the prediabetic group. However, in prediabetic subjects, a positive correlation between TAS and WC and a negative correlation between TAS and HDL-C were found. In addition, in PRE group, TBARS correlated positively with fasting glucose and HbA1c and negatively with age and BMI, whereas we did not observe such a correlation in the normoglycemic elderly group.

## 4. Discussion

Oxidative stress and failure of protein repair are one of the most discussed abnormalities in the aging process—both at the cellular and tissue levels [22, 23].

In the present study we investigated only elderly persons with or without prediabetic states to find out that oxidative stress and its markers depend not only on aging but also on hyperglycemia and its complications. Antioxidant defense systems, both located in the intracellular and extracellular spaces, are actively involved against reactive oxygen species, which are continuously generated in the body due to normal metabolism and disease. Studies concerning patients with late diabetic complications [24] or without them [25] have revealed a decrease in antioxidant defenses and an increase in oxidative damage markers. The authors of the present study investigated antioxidant status at the very early stages of hyperglycemia and found lower SOD-1 activity and plasma TAS in prediabetic elderly persons in comparison with normoglycemic ones.

Nakhjavani and colleagues suggested that the chronicity of DM promotes lipid peroxidation and malondialdehyde production, independent of glycemic control and antioxidant activity [26]. In our study we did not find any differences in the TBARS (as investigated for lipid peroxidation products) between normoglycemic and prediabetic elderly people, whereas SOD-1 and TAS were lower in prediabetic ones. Thus, we suggest that antioxidant capacity is the first marker which declines in prediabetic elderly people. Kumawat and colleagues concluded that there is enhanced oxidative stress and decreased antioxidant defense in geriatrics as compared to younger counterparts [27]. However, their elderly group had highly increased total cholesterol, triacylglycerols, LDL-cholesterol, and decreased HDL-cholesterol. We investigated only elderly population with lipid profile within references but with or without hyperglycemia and thus we suggest that decreased antioxidant capacity is rather due to hyperglycemia than aging itself.

It is important to note that longer duration of hyperglycemia and chronic diabetes complications are associated with older age [28]. Our findings showed strong positive correlation between SOD-1 and TAS in elderly normoglycemic subjects, whereas in prediabetic ones there is deactivation between intra- and extracellular antioxidative state (Table 3).

The authors of the present work found an interesting negative correlation between TAS and HDL-C in prediabetic elderly people, independent of other metabolic factors (Table 7). The linear changes of plasma HDL-C concentration may accompany or even supplement 28% of plasma TAS variability. This suggests the complementarity of these two important antioxidant factors in elderly patients with high risk for T2DM. It also supports the current suggestion about HDL function in humans, what was pointed out not only in hyper-LDL-C patients but in normal low density lipoprotein levels patients as well [29].

A very interesting work was published by Bandeira and colleagues, and they found correlation between lipid peroxidation and diabetes mellitus irrespective of the presence of hypertension [30]. In the present work the multiple regression analysis showed negative correlation between TAS and fasting glycemia in those without hypertension, what suggests that fasting glucose in 80% accompanies TAS in the preservation of development of hypertension and positive correlation between TBARS and fasting glycemia in those with developed hypertension, which is the next point of developing chronic complications in elderly prediabetic patients. Thus, we suggest that disturbances in the oxidative-antioxidative status may serve as very early markers of chronic complications of hyperglycemia.

*Limitation of the Study*. Although there is much that remains to be done, our work generates important findings in the field of antioxidant capacity among elderly population. We confirm that there are some limitations of this study. The main limitation is small elderly group, but it is hard to find elderly subjects without complaints, with no acute and/or chronic diseases, using no medication or supplements. Future research would have been more convincing if the researchers would have more elderly subjects with the very early hyperglycemia state both impaired glucose tolerance and impaired fasting glycemia.

## 5. Conclusions

In elderly patients metabolic factors differ among prediabetic and normoglycemic patients leading to disturbances in oxidative-antioxidative state. Erythrocyte SOD-1 activity and plasma TAS are lower in elderly prediabetics in comparison with normoglycemic cases revealing deactivation of antioxidative capacity by hyperglycemia in elderly patients. In elderly prediabetic subjects, TBARS did not differ significantly in comparison with control group, indicating early oxidative stress. Thus, SOD-1 and TAS are suggested to be the very early biomarkers in the course of hyperglycemic complication among prediabetic elderly people. Identification of pathomechanisms involved in disturbances of carbohydrate metabolism in the course of early diabetes stages enables the explanation of chronic diabetic complications leading to optimization of the treatment in elderly hyperglycemic cases.

## Figures and Tables

**Table 1:** The interpretation of oral glucose tolerance test (OGTT) adapted from [20].

Categories of glycemia during OGTT	Plasma glucose concentration	
	Fasting (at 0 min)	At 120 min
Normal glucose tolerance (NGT )	<5.6 mmol·L^−1^ <100 mg·L^−1^	<7.8 mmol·L^−1^ <140 mg·L^−1^
High risk of diabetes (prediabetes, PRE)		
Impaired fasting glycemia (IFG)	5.6–6.9 mmol·L^−1^ 100–125 mg·L^−1^	<7.8 mmol·L^−1^ <140 mg·L^−1^
Impaired glucose tolerance (IGT)	<7.0 mmol·L^−1^ <126 mg·L^−1^	7.8–11.0 mmol·L^−1^ 140–199 mg·L^−1^
Diabetes mellitus (DM)	<7.0 mmol·L^−1^ <126 mg·L^−1^	≥11.1 mmol·L^−1^ ≥200 mg·L^−1^

**Table 2:** The characteristics of the study groups. Data are presented as mean ± SD for Gaussian distribution and median with interquartile range for the non-Gaussian distribution.

	Total *n* = 46	NGT *n* = 18	PRE *n* = 28
Age (years)	70.0 (67.0–74.0)	69.0 (66.0–73.0)	71.0 (67.0–75.0)
BMI (kg·m^−2^)	28.0 (26.0–30.8)	26.0 (24.0–28.5)	29.3 (26.5–34.2)*
WC (cm)	92.0 (83.0–98.0)	84.5 (78.0–92.0)	94.0 (88.0–104.0)*
FAT (%)	37.2 +/− 15.2	34.4 +/− 18.4	38.6 +/− 12.8
SBP (mmHg)	140.0 (130.0–145.0)	132.5 (125.0–145.0)	140.0 (135.0–145.0)
DBP (mmHg)	80.0 (75.0–90.0)	85.0 (80.0–90.0)	80.0 (70.0–85.0)
G0′ (mmol·L^−1^)	5.71 (5.17–6.35)	5.13 (4.97–5.39)	6.19 (5.83–6.44)
G120′ (mmol·L^−1^)	6.67 (5.56–7.50)	5.62 (5.03–6.67)	7.22 (5.70–8.5)
Ins 0′ (mU·L^−1^)	30.83 (16.39–34.66)	29.25 (16.39–31.93)	32.85 (16.54–37.44)
Ins 120′ (mU·L^−1^)	60.41 (27.65–90.97)	62.68 (49.29–81.66)	56.38 (27.51–120.64)
HbA_1c_ (%)	5.99 (5.60–6.40)	5.80 (5.60–6.10)	6.20 (5.50–6.50)
T-C (mmol·L^−1^)	5.13 (4.64–5.70)	5.71 (4.79–5.78)	5.05 (4.61–5.67)
TAG (mmol·L^−1^)	0.94 +/− 0.75	1.24 +/− 0.59	1.32 +/− 0.85
HDL-C (mmol·L^−1^)	1.66 (1.41–1.79)	1.71 (1.48–1.81)	1.65 (1.29–1.78)
LDL-C (mmol·L^−1^)	2.90 (2.49–3.46)	3.11 (2.58–3.54)	2.79 (2.45–3.24)
SOD-1 (U·gHGB^−1^)	1049.9 (886.7–1257.9)	1208.6 (1002.4–1623.0)	980.4 (841.7–1137.2)*
TAS (mmol·L^−1^)	1.338 (1.228–1.510)	1.341 (1.280–1.717)	1.303 (1.154–1.401)*
TBARS (*μ*mol·L^−1^)	2.082 (1.737–2.416)	1,902 (1.722–2.088)	2.189 (1.770–2.952)

*Significant difference, as compared to NGT group.

NGT: normal glucose tolerance, PRE: prediabetes states, FAT: percentage of body fat, BMI: body mass index, WC: waist circumference, SBP: systolic blood pressure, DBP: diastolic blood pressure, G0′: fasting glucose, G120′: glucose at 120 min of oral glucose tolerance test (OGTT), Ins 0′: fasting insulin, Ins 120′: insulin at 120 min of OGTT, HbA_1c_: glycated hemoglobin, T-C: total cholesterol, TAG: triacylglycerols, HDL-C: high density lipoprotein cholesterol, LDL-C: low density lipoprotein cholesterol, SOD-1: Cu-, Zn-superoxide dismutase, TAS: total antioxidant status, and TBARS: thiobarbituric acid-reacting substances.

**Table 3:** The correlations between oxidative stress markers and clinical and biochemical parameters in the studied subjects.

Variables	NGT						PRE					
	SOD-1		TAS		TBARS		SOD-1		TAS		TBARS	
	*R*	*P*	*R*	*P*	*R*	*P*	*R*	*P*	*R*	*P*	*R*	*P*
Age	−0.379	0.120	−0.258	0.301	0.341	0.166	0.245	0.218	0.024	0.907	**−0.659**	**<0.001 **
BMI	0.229	0.360	−0.012	0.961	0.198	0.430	0.192	0.924	0.115	0.567	**−0.437**	**0.023**
WC	−0.174	0.491	−0.215	0.391	0.337	0.172	0.209	0.295	**0.398**	**0.040**	−0.164	0.413
FAT	−0.034	0.893	−0.106	0.675	0.242	0.332	0.200	0.585	0.042	0.835	−0.366	0.061
SBP	−0.028	0.912	−0.203	0.418	0.229	0.360	−0.159	0.429	0.113	0.574	0.057	0.777
DBP	0.017	0.946	−0.105	0.677	−0.044	0.863	−0.068	0.738	0.185	0.357	−0.204	0.307
G0′	0.189	0.453	0.130	0.607	0.032	0.900	−0.318	0.106	−0.080	0.693	**0.407**	**0.035**
G120′	−0.057	0.823	−0.118	0.639	0.358	0.144	0.062	0.759	−0.029	0.886	0.314	0.110
Ins 0′	−0.154	0.542	−0.349	0.155	0.120	0.636	−0.052	0.794	**0.422**	**0.025**	**−0.403**	**0.033**
Ins 120′	−0.135	0.593	−0.244	0.328	0.253	0.311	0.214	0.273	0.104	0.597	0.177	0.368
HbA_1c_	−0.140	0.579	−0.003	0.990	0.351	0.153	−0.056	0.779	−0.093	0.645	**0.503**	**0.007**
T-C	0.320	0.195	0.272	0.275	−0.012	0.961	0.055	0.786	−0.200	0.317	0.186	0.354
TAG	0.020	0.936	0.041	0.872	0.118	0.642	−0.255	0.199	0.040	0.843	0.082	0.684
HDL-C	0.174	0.489	0.236	0.345	−0.170	0.499	−0.029	0.885	**−0.568**	**0.002**	−0.194	0.332
LDL-C	0.376	0.123	0.234	0.349	−0.141	0.576	0.173	0.387	−0.025	0.902	0.222	0.266
SOD-1	—	—	**0.864 **	**<0.001**	−0.416	0.086	—	—	0.225	0.258	−0.052	0.795
TAS	**0.864**	**<0.001 **	—	—	−0.178	0.478	0.225	0.258	—	—	−0.221	0.267
TBARS	−0.416	0.086	−0.178	0.478	—	—	−0.052	0.795	−0.221	0.267	—	—

NGT: normal glucose tolerance, PRE: prediabetes states, FAT: percentage of body fat, BMI: body mass index, WC: waist circumference, SBP: systolic blood pressure, DBP: diastolic blood pressure, G0′: fasting glucose, G120′: glucose at 120 min of oral glucose tolerance test (OGTT), Ins 0′: fasting insulin, Ins 120′: insulin at 120 min of OGTT, HbA_1c_: glycated hemoglobin, T-C: total cholesterol, TAG: triacylglycerols, HDL-C: high density lipoprotein cholesterol, LDL-C: low density lipoprotein cholesterol, SOD-1: Cu-, Zn-superoxide dismutase, TAS: total antioxidant status, and TBARS: thiobarbituric acid-reacting substances.

**Table 4:** The significant correlations in the multiple regression analysis between oxidative stress markers and clinical and biochemical parameters in the model A, including age, BMI, waist, and FAT.

	*B*	Beta	*P*	*r*	*R* ^2^
TBARS and age All subjects	3.6400	−0.02746	0.0462	−0.3008	0.1175
TBARS and waist Males	2.4462	0.02728	0.0215	0.3240	0.5515
TBARS and age G0′ = 1	5.9268	−0.03448	0.0402	−0.4909	0.3053
TBARS and age Pre = 1	6.1224	−0.03550	0.0347	−0.5050	0.3379
SOD-1 and BMI G0′ = 0	3235.9562	59.4009	0.0405	0.2569	0.4653
SOD-1 and BMI PRE = 0	2522.5913	66.3857	0.0340	0.4116	0.4653
TAS and FAT G0′ = 0	3.0917	−0.008491	0.0433	−0.4332	0.4150

PRE = 0: no prediabetes states, PRE = 1: prediabetes states, G0′ = 0: no hyperglycemia, G0′ = 1: fasting hyperglycemia, FAT: percentage of body fat, BMI: body mass index, SOD-1: Cu-, Zn-superoxide dismutase, TAS: total antioxidant status, and TBARS: thiobarbituric acid-reacting substances.

**Table 5:** The significant correlations in the multiple regression analysis between oxidative-antioxidative status and clinical and biochemical parameters in the model B, including HbA_1c_, G0′, and G120′.

	*B*	Beta	*P*	*r*	*R* ^2^
TBARS and HbA_1c_ All subjects	−2.4365	0.4511	0.0225	0.5422	0.4118
TBARS and HbA_1c_ Females	−2.6736	0.5289	0.0479	0.6294	0.4893
TBARS and HbA_1c_ AH = 1	−2.4637	0.4848	0.0369	0.4812	0.3533
TBARS and G0′ AH = 1	−2.4637	0.01756	0.0255	0.4844	0.3533
TBARS and G0′ All subjects	−2.4365	0.01504	0.0317	0.4959	0.4118
SOD-1 and G0′ Females	2473.4391	−15.0145	0.0293	−0.4508	0.2075
TAS and G0′ Females	1.8850	−0.01307	0.0159	−0.4636	0.2567
TAS and G0′ AH = 0	3.5926	−0.04342	0.0184	−0.7862	0.8063

AH = 0: no arterial hypertension, AH = 1: with arterial hypertension, G0′: fasting glycemia, HbA_1c_: glycated hemoglobin, SOD-1: Cu-, Zn-superoxide dismutase, TAS: total antioxidant status, and TBARS: thiobarbituric acid-reacting substances.

**Table 6:** The significant correlations in the multiple regression analysis between oxidative-antioxidative status and the clinical and biochemical parameters in the model C including SBP, DBP, age, BMI, FAT, and waist.

	*B*	Beta	*P*	*r*	*R* ^2^
TBARS and SBP All subjects	3.7117	0.01727	0.0205	0.2325	0.3167
TBARS and SBP Females	3.4882	0.01664	0.0470	0.2276	0.3941
TBARS and SBP AH = 1	4.5396	0.01606	0.0479	0.2090	0.4041
TBARS and DBP All subjects	3.7117	−0.02932	0.0051	−0.2777	0.3167
TBARS and DBP Females	3.4882	−0.03155	0.0221	−0.3767	0.3941
TBARS and DBP AH = 1	4.5396	−0.02766	0.0108	−0.4208	0.4041
TBARS and age AH = 1	4.5396	−0.03200	0.0485	−0.3471	0.4041
SOD and BMI G0′ = 0	2678.6219	64.3659	0.0473	0.2569	0.4946

AH = 0: no arterial hypertension, AH = 1: with arterial hypertension, G0′ = 0: no fasting hyperglycemia, G0′ = 1: fasting hyperglycemia, BMI: body mass index, SBP: systolic blood pressure, DBP: diastolic blood pressure, SOD-1: Cu-, Zn-superoxide dismutase, TAS: total antioxidant status, and TBARS: thiobarbituric acid-reacting substances.

**Table 7:** The significant correlations in the multiple regression analysis between studied oxidative-antioxidative status and clinical and biochemical parameters in the model D including TC, HDL-C, and TAG (LDL-C not included as a derivative of analyzed variables).

	*B*	Beta	*P*	*r*	*R* ^2^
SOD and TC All subjects	811.6939	3.6717	0.0421	0.2094	0.1134
TAS and HDL G0′ = 1	1.8910	−0.009533	0.0245	−0.5293	0.2806
TAS and HDL PRE = 1	1.9046	−0.009167	0.0309	−0.5320	0.2839

G0′ = 0: no fasting hyperglycemia, G0′ = 1: fasting hyperglycemia, PRE = 0: no prediabetes, PRE = 1: prediabetes, TC: total cholesterol, HDL-C: high density lipoprotein cholesterol, SOD-1: Cu-, Zn-superoxide dismutase, and TAS: total antioxidant status.
